# Low-loss skimming waveguides with controllable mode leakage for on-chip saturable absorbers

**DOI:** 10.1515/nanoph-2023-0049

**Published:** 2023-05-31

**Authors:** Yi Yang, Lijing Zhong, Yudong Cui, Yuying Wang, Daoyuan Chen, Kuen Yao Lau, Xiaofeng Liu, Zhijun Ma, Giuseppe Barillaro, Zhi Chen, Jianrong Qiu

**Affiliations:** State Key Laboratory of Modern Optical Instrumentation, College of Optical Science and Engineering, Zhejiang University, Hangzhou, 310027, China; Zhejiang Lab, Hangzhou, 311121, China; School of Materials Science and Engineering, Zhejiang University, Hangzhou, 310027, China; Dipartimento di Ingegneria dell’Informazione, Università di Pisa, Pisa, Toscana, Italy

**Keywords:** carbon nanotube, femtosecond laser direct writing, optical waveguide, Q-switched pulse laser, saturable absorber

## Abstract

Emerging 3D photonic circuits would greatly benefit from the ability to integrate skimming waveguides with low loss and controllable inscription depth into photonic circuits. These waveguides allow for the interaction of guiding light directly with external modulation signals and enable programmable photonic circuits. Here, we report the fabrication of a novel photonic-lattice-like skimming waveguide (PLLSW) using femtosecond laser writing. Our method enables fine control of cross-sectional symmetry and writing depth of waveguides, achieving a minimum depth of 1 μm and a low insertion loss of 1 dB. Based on the PLLSW, we demonstrate on-chip light modulation by designing an evanescent-field-type saturable absorber through the coupling of a carbon nanotube film with the PLLSW, which exhibits saturation intensity from 20 to 200 MW/cm^2^ through the balanced twin-detector measurement. The strong nonlinear optical response of the PLLSW-based saturable absorber is further exploited to drive a Q-switched pulse laser at 1550 nm based on a fiber laser cavity. Our work demonstrates an effective method to integrate nonlinear optical materials into a glass chip for all-optical switching based on 3D waveguides, which holds great potential for the construction of large-scale programmable photonic circuits in the future.

## 1 Introduction

Integrated optical waveguide with low loss has seen its broad applications, including, quantum optics [1, 2], integrated photonic chips [3, 4], topological photonics [5, 6], and programmable photonic circuits [7, 8]. Traditionally, the waveguides are fabricated on basis of silica-on-silicon technology [9], faced with problems of low manufacturing efficiency, high expense, and easy-affecting by external environment due to only 2D planar waveguides can be achieved, limiting the development of programmable photonic circuits.

Comparably, femtosecond laser direct-writing (FLDW) is a rapid prototyping technique for writing large-scale complex photonic circuits in a single step [10, 11], which can greatly simplify manufacturing procedures and shorten the technical iteration cycle from a few months of conventional lithography to a few hours or even less [12]. With the advantage of flexible three-dimensional (3D) manufacturing, FLW has been widely applied for fabricating 3D optical waveguides components in transparent materials including glass [13–16] and crystal [13, 17, 18]. However, traditional FLW structures are generally deeply beneath the glass surface; therefore, they are insensitive to external environment changes and thus difficult to be tuned by an external signal. In order to dynamically control waveguides properties and realizing programmable photonic circuits, it is crucial to couple the waveguide closely with an optically active medium (such as graphene [19, 20] and indium tin oxide [21]) so that the guided light could be modulated through the dynamic optical response of the material. In this sense, a near-surface waveguide is a trivial solution to make the light in FLDW waveguides directly interacting with external environment through the evanescent wave, and realizing the light modulation through the external signal such as temperature gradient [22, 23] and refractive index change [24–26]. However, waveguides inscribed close to the glass surface usually suffer from highly non-symmetric cross-section and lower refractive index contrast compared with the deeply buried ones, due to the air–glass interface aberration and surface ablation [27, 28].

To overcome this problem, in this work, we put forward a new multi-scan laser direct-writing technique [29] and demonstrate a novel photonic-lattice-like skimming waveguide (PLLSW). Specifically, we design and fabricate a fiber-compatible PLLSW with a diameter of 10 μm by carefully arranging scanning traces with a single modified cross-section of only 1.2 × 1.5 μm^2^, achieving a low insertion loss of 1 dB. This method allows for construction of waveguide with high spatial resolved cross-section, enabling variable cross-section and avoiding laser-ablation of glass surface. Herein, we realize a fine control of PLLSWs depth over a wide range from 1 μm to 10 μm, which enables the control of mode leakage. Furthermore, based on evanescent field interaction, we design an on-chip waveguide-coupled saturable absorber (SA) by integrating a single-wall carbon nanotube film onto the PLLSW, which exhibits strong nonlinear optics (NLO) absorption in the near-infrared (NIR) region and enables Q-switched pulse generation based on a Er^3+^-doped fiber laser. Our work delineates an effective method to integrate nonlinear optical media with a monolithic glass chip, allowing for all-optical control in communication window, which is promising for large-scale programmable photonic circuits in the future.

## 2 Results and discussions

**Figure 1:**
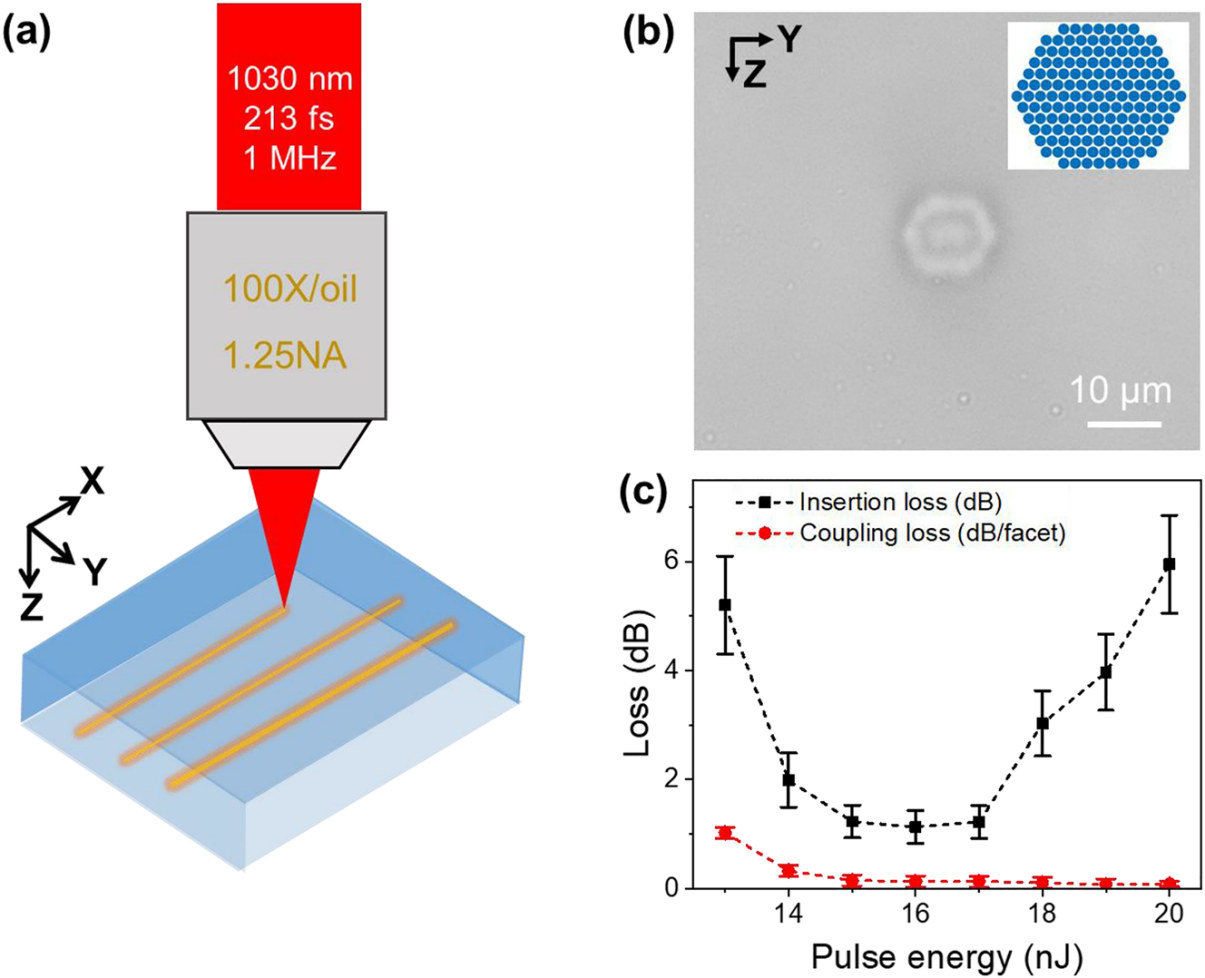
Scheme of FLDW strategy and optical characterizations of the PLLSW. (a) Experimental setup for the FLDW. (b) Microscope YZ-cross-sectional image of a PLLSW fabricated with pulse energy of 16 nJ, with schematic diagram inset. (c) Optical loss of PLLSW, including insertion loss (black line) and coupling loss (red line), as a function of writing pulse energy.

The experiment setup for FLDW is shown in Figure 1(a). A diode-pumped Yb:KGW laser (PHAROS PH2, Light Conversion) provides 213 fs pulses at a center wavelength of 1030 nm with a repetition rate of 1 MHz. The laser beam is focused below the surface of borosilicate glass (Corning Eagle XG) samples. A high-precision XYZ translation stage (Aerotech, A3200) is used to control the 3D movement of the glass sample at the set routine and speed. After laser writing, both ends of the sample are cut and polished. To avoid laser ablation of the glass surface, we use a relatively low power of 15–17 nJ and an objective lens with high numerical aperture (NA) of 1.25 to tightly focus the laser beam (Figure 1(a)), and the resultant single writing area can be down to ∼1.5 × 1.2 μm^2^ (Supplementary Figure S1). However, the single-write trace geometry does not match the core diameter of a single-mode fiber operating at 1550 nm (SMF-28 fiber with a core diameter of 8.2 μm). To tailor the waveguide cross-section and reduce the coupling loss caused by the mode field mismatch, we use multi-scan method by designing a fiber-compatible waveguide, i.e., PLLSW. The PLLSW is based on a hexagonal-shaped stack with diameter of ∼10 μm consisting a bundle of writing traces. This structure yields a centrosymmetric waveguide. The row and column spacing between adjacent traces is 0.6 μm and 0.7 μm, respectively.

A microscope image of the PLLSW taken from YZ-section is shown in Figure 1(b), we can observe a bright hexagon with a diameter of ∼10 μm, in accordance with the dimension of the designed waveguide. This is due to the positive index change of the writing area that constrains the illumination in the bright area, therefore the PLLSW is a type-I waveguide [30]. However, one may notice a multilayer structure consisting of a dark stripe wrapping around the bright central region, which is possibly due to the influence of higher-order modes in visible transmitted light. Actually, the PLLSW pattern observed by the microscope is dependent on the writing laser power (Supplementary Figure S2). Except for the moderate writing pulse energy (15–17 nJ), the PLLSW written by using lower power (<15 nJ) is blurred in micrographs, in comparison with that written by higher energy is bright, indicating that higher laser power effectively induce a larger refractive index change.

To find the appropriate writing pulse energy, a series of waveguides were fabricated with a scanning speed of 5 mm/s. The waveguide was characterized with light of wavelength 1550 nm via single-mode fibers butt-coupled to the waveguides. The insertion loss of the PLLSW waveguides is calculated from the power carried by input fiber divided by the power output from the PLLSW waveguide. To obtain the near-field mode profile of the PLLSW waveguide, we replace the output fiber by a long-focal-distance lens (Mitutoyo M Plan Apo NIR 50X, 0.42NA). Then, the near-filed mode profile was imaged by a beam profiler (CMOS-1201-IR, CINOGY) after adjusting the position of the lens.

The dependence of insertion loss on writing pulse energy is shown in Figure 1(c) (black line). For writing pulse energy less than 15 nJ, the insertion loss decreases as the writing power increases. This is because the refractive index changes increase as the writing power increases. Therefore, the mode field of PLLSW becomes smaller, and the coupling loss (red line in Figure 1(c)) calculated by the mode field overlapping integration (see [31] and Supplementary text) shows similar trend as that of the insertion loss. Besides, the lower index change makes waveguides more susceptible to micro-bending loss and the insertion loss. By contrast, the insertion loss increases as the writing power rises for writing pulse energy higher than 17 nJ. A higher writing pulse energy generate a larger modified area and, even worse, introduce voids and cracks (Supplementary Figure S2), which break the uniformity of the PLLSW and cause additional scattering loss. Therefore, the PLLSW with insertion loss as low as 1 dB can be achieved by optimizing the pulse energy within 15–17 nJ. The PLLSWs mentioned later in this work are fabricated under the same conditions.

**Figure 2:**
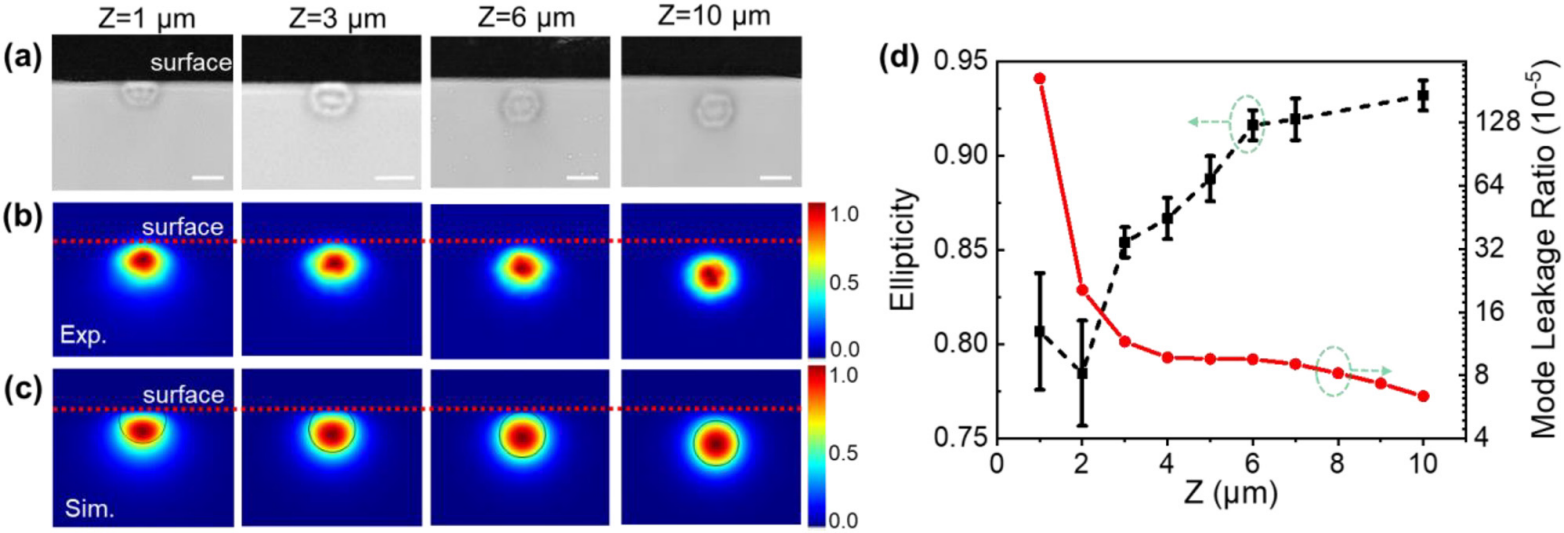
PLLSW at different depths from the glass surface. (a) Microscope images of the PLLSWs cross-sections at depths of 1–10 μm from the center position of the waveguides, and (b) corresponding mode field distributions and (c) mode simulation results of the PLLSWs at 1550 nm. Red dashed lines mark the interface between air and glass. (d) The measured mode ellipticity and calculated mode leakage ratio of PLLSWs. Scale bars are 10 μm.

To control the mode leakage of the PLLSWs, we fabricate different PLLSWs with depth changing from 1 μm to 10 μm. As evidenced in Figure 2(a) and Supplementary Figure S3, the PLLSWs keep the designed structure without any surface ablation or destruction even if parts of PLLSW leak out of the surface, indicating the multi-scan writing is a robust approach to fabricate the PLLSW closed to the surface. To validate the controllability of the mode leakage of the PLLSWs, mode profile was detected, as showed in Figure 2(b). For the deep PLLSW (e.g., *Z* = 10 μm), the mode profile is circle-like and its ellipticity (minor axis divided by major axis) is 0.932 (Figure 2(d)). In this condition, the air layer has a weak influence on the mode profile. Comparably, when the waveguides are close to the surface, the mode profile changes from circle to an asymmetric ellipse shape, and the area along the negative *z*-axis above the center of the mode profile reduces. It is due to the huge refractive index contrast between air and glass, such that light attenuates rapidly in air even if the waveguide is near the surface. It is worth noting that the mode field size of waveguide at the depth of *Z* = 1 μm becomes larger than the one at the depth of *Z* = 2 μm, while the waveguide cross-section size is smaller. This indicates that the normalized cut-off frequency of the waveguide is close to zero [32], and such waveguide is vulnerable to macro-bend which might be created by defects in laser writing [33]. Actually, we can fabricate a PLLSW at a minimal depth of *Z* = −4 μm from center of the waveguide, without any surface ablation or damages. The optical simulation for the guiding mode profile of the PLLSW waveguide was processed by the commercial COMSOL software. In simulation, we use a circle model with uniform index to represent the waveguide structure, and the refractive index difference between waveguide and pristine glass is set at 0.003. The simulation results at each depth (shown in Figure 2(c)) are consistent with the mode profiles taken from experimental data, confirming the uniformity of waveguide index variation. The mode leakage ratio is defined as the ratio of the peak intensity of the mode field leaked into the air to that of the waveguide mode, showing that the mode leakage decreases rapidly with the increase of the waveguide depth (Figure 2(d)). Hence, we believe that the low-loss PLLSW written by this new technique is uniform and free of cracks and has high mode leakage controllability, which has great potential for application in on-chip integrated photonic devices.

**Figure 3:**
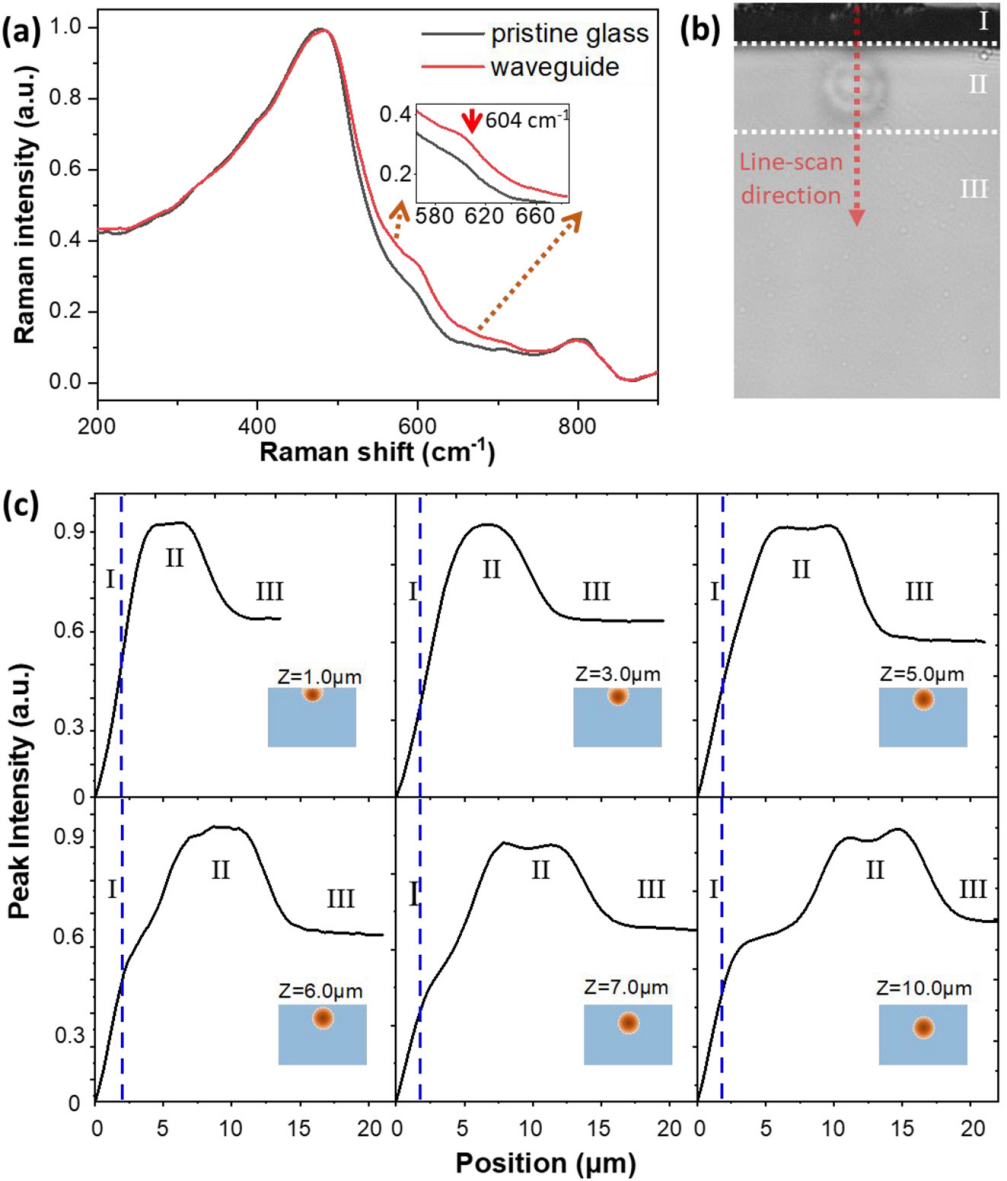
Refractive index distribution of PLLSW waveguides featured by Raman measurements. (a) Raman spectra of the PLLSW waveguide and pristine glass. Inset, enlarged feature peak of defect band. (b) Line-scan area along *Z*-direction across the glass surface and center of waveguides. (c) Raman intensity of the line-scan at 604 cm^−1^ at different depths. Dash lines represent the edge of glass.

To further verify the change of refractive index in the PLLSWs, a confocal Raman microscope (Renishaw) was employed to characterize the microstructures. The Raman spectra of the PLLSW and pristine glass are shown in Figure 3(a). A narrow band around 604 cm^−1^ appears after FLDW, which is known as defect band related to the three-membered rings in the glass [34]. Importantly, the Raman peak at around 604 cm^−1^ has been related with the positive index change [34–36], therefore, the Raman intensity at the peak of 604 cm^−1^ is used as a quantitative representation of waveguide refractive index change. We recorded Raman mapping (line-scan, Figure 3(b)) of different depth PLLSWs along *z*-axis at 604 cm^−1^, as shown in Figure 3(c). The Raman intensity profile is consistent with the cross-section of the designed waveguides, indicating a positive index change area in the region of the PLLSW. Each Raman mapping can be divided into three distinct regions including: (I) a region with near zero signal intensity, which ascribed to air; (II) the region with high intensity that in region, which represents the positive index change area; (III) the region with medium intensity, which is ascribed to the pristine glass. Because the radius of the laser spot used in the Raman microscope is ∼2 μm, the Raman mapping cannot clearly identify the boundary of the fabricated waveguide. Although the results can not reflect the sharp structure change, it is quite enough to confirm the index change in the PLLSW formed in glass after FLDW. For the PLLSW fabricated at depth of *Z* < 5 μm, the width of the region II narrows with some of traces above the glass surface. For depth *Z* > 5 μm, the major difference of the PLLSWs is the vertically displacement of region II. In all the results, the Raman intensity of region II is higher than that in region III by 30 %, indicating the index change is uniform and consistent with the waveguide cross-section. Thus, we develop a method to fabricate PLLSW with low loss and high uniformity. By changing the writing depth, the PLLSWs have controllable mode field leakage, which is useful in many fields, including on-chip integrated photonic circuit and optical switching [7, 37].

To make use of the evanescent field interaction of the PLLSW, we experimentally realized an SA device consisting of the PLLSWs at depth of 1–10 μm integrated with SWCNT/PVA hybrid film [38]. The SWCNT/PVA hybrid film was prepared by mixing a suspension of SWCNT and sodium dodecyl benzene sulfonate (SDBS) into a PVA solution as a composite. The SWCNT is a long prototypical quantum-confined 1D material which is frequently designed with tube diameter of ∼1 nm by rolling monolayer graphene [39]. The optical absorption of the SWCNT is determined by its tube diameter which provides different optical bandgap according to the Kataura plot [40]. For instance, the SWCNT could be designed with a tube diameter ranging from 0.7 to 1.5 nm, which corresponds to bandgap energy of 1.2 to 0.6 eV with an emission in near-infrared wavelength from 1 to 2 μm. Apart from that, the third-order nonlinear susceptibility (χ^(3)^) of the SWCNT was measured to be ∼10^−7^ e.s.u. for its imaginary part (Im) through a pump-probe setup [41]. The Im(χ^(3)^) is related to the nonlinear absorption coefficient (*α*_NL_) according to:

(1) $$Im\chi^{\left(3\right)}=\left[\left(10^{-7}c\lambda n^{2}\right)/96\pi^{2}\right]\cdot \alpha_{\text{NL}}$$

where *c* is the speed of the light in vacuum, *λ* is the wavelength of incident light, and *n* is the refractive index of the SWCNT [42]. The *α*_NL_ depends on the optical intensity or radiant flux either on the instantaneous intensity or to the intensities in the near past. At higher intensity, the reduced α_NL_ corresponds to the saturable absorption. The saturable absorption is employed for pulsed laser generation through either passive mode-locking or Q-switching regimes. A host polymer, i.e. water-soluble PVA was used to prepare a stable and high concentration aqueous SWCNT dispersion [43]. The resulting SWCNT/PVA hybrid film is mechanically strong in composite form with smooth surface. A smooth SWCNT/PVA hybrid film was prepared in this work as depicted in the inset (area with dark-grey substance) of Figure S4. The SWCNT/PVA hybrid film was employed for the studies of saturable absorption and pulsed laser generation.

**Figure 4:**
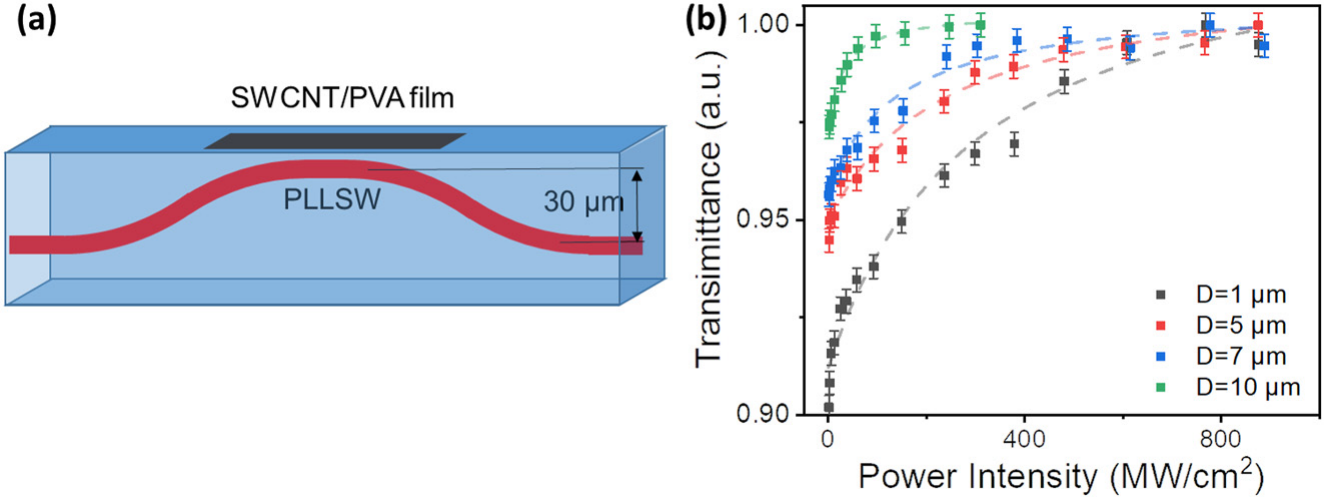
SWCNT/PVA film integrated PLLSW saturable absorber. (a) Design scheme of S-bend waveguide. (b) Dependence of the transmittance of the PLLSWs integrated with SWCNT/PVA film on the power density of input laser.

To precise control the interaction length between the PLLSW and SWCNT/PVA film, we design and write an S-bend waveguide with changeable interaction length (Figure 4(a)). The optimized interaction length is around 1.0 mm. To investigate the nonlinear optical properties of the chip-integrated SA device (Figure S4, insert), we employed a balanced twin-detector measurement system (Figure S4) to measure the power-dependent transmission of the device. Figure 4(b) shows the dependence of the transmittance of the PLLSW integrated with SWCNT/PVA film on the input power intensity. All the integrated devices with the PLLSW at depth of 1–10 μm show saturable absorption feature, which can be well fitted by a two-level mode [44]:

(2) $$T\left(I\right)=A\exp\left(\frac{-\Delta T}{1+I/I_{\text{sat}}}\right)$$

Where, *T* represents the transmission of device, *I* is the incident optical power, Δ*T* stands for the modulation depth, and *I*_Sat_ is the saturation power. The experiment data can be well fitted by using this theoretical mode, indicating that the saturable absorption behavior happens in the PLLSW integrated with SWCNT/PVA film. The maximum modulation depth of 10 % is obtained for the PLLSW fabricated at the depth of 10 μm and the minimum saturation power of 22.1 MW/cm^2^ is obtained for the waveguide depth of 1 μm. Notably, both of the *I*_Sat_ and Δ*T* increase as the depth reduces. As more leaked light interacting with the SWCNT/PVA film, the saturable absorption effect becomes stronger, resulting in the increase of Δ*T*. The possible reason for the increase of *I*_Sat_ is the enhancement of non-saturable absorption with the reduction in the PLLSW depth.

**Figure 5:**
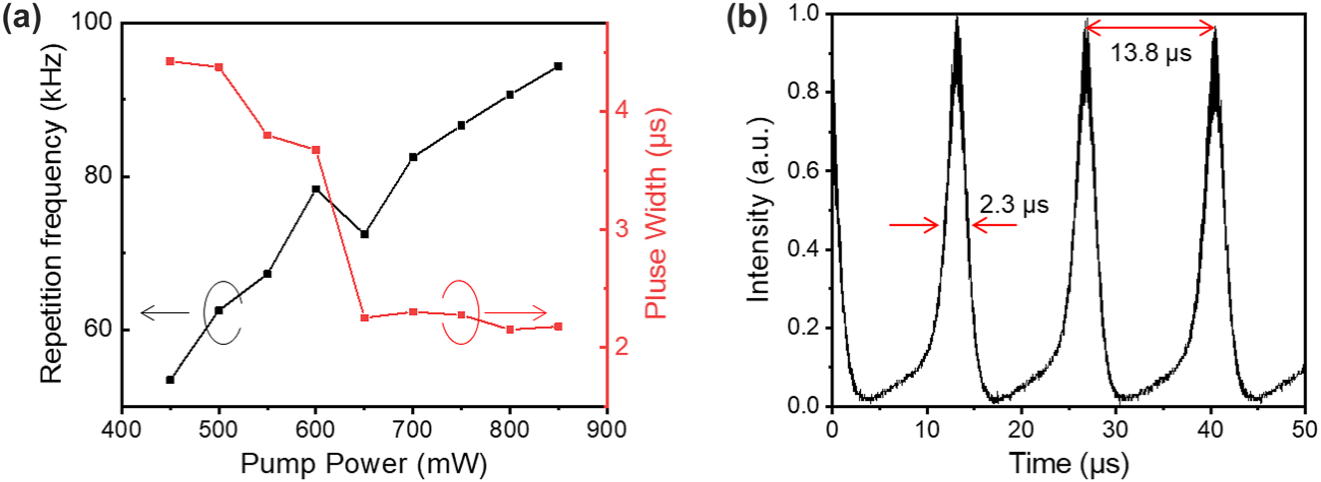
Demonstration of the PLLSW waveguide integrated with the SWCNT/PVA film for Q-switched pulse generation based on an Er^3+^ doped fiber laser. (a) Repetition rate and pulse width against pumping power. (b) Pulse train at a pumping power of 650 mW.

Based on the developed SA, we make a Q-switched fiber pulse laser (Supplementary Figure S5), by integrating the above waveguide-coupled SA into an Er^3+^-doped fiber laser operating at 1.55 μm. As shown in Figure 5(a), the repetition rate evolves from 53.5 to 94.3 kHz, and the pulse width declines from 4.425 to 2.175 μs with the pump power increasing from 400 to 900 mW. Figure 5(b) depicts the behavior of the Q-switched laser at the pumping power of 650 mW, with a repetition period of 13.8 μs and a pulse width of 2.3 μs. This result indicates that the PLLSW could be a versatile platform for integrating NLO materials that allows for on-chip all-optical switching for photonic devices.

Thus, we have demonstrated on-chip light modulation by designing an evanescent-field-type saturable absorber through the coupling of a carbon nanotube film with the PLLSW. The strong nonlinear optical response of the PLLSW-based saturable absorber is further exploited to drive a Q-switched pulse laser. Our work delineates an effective method to integrate nonlinear optical materials into a glass chip for all-optical switching based on low-loss skimming waveguides, which can be directly integrated into FLDW 3D photonic circuits and compatible with optical fibers, and has the advantages of flexible control of leaky modes and higher laser damage threshold over traditional waveguide modulators including D-shaped fiber [45] and micro/nanofiber [46]. In addition to the nonlinear optical modulation exemplified in this work, PLLSWs also hold great promise for realizing a wide variety of integrated photonic devices by dressing them with functionalized materials and structures such as plasmonic nanoparticles [47], 2D materials [48], and metasurface structure [49]. Recently, we noticed similar efforts to develop near-surface FLDW waveguides with controllable depth, with the aim of making FLDW waveguides sense external changes for developing sensors [50], and reducing crosstalk between FLDW waveguides for building configurable photonic circuits [51]. In this sense, the proposed PLLSW provides a miniature waveguide-optic platform with high compactness and great versatilities, which can be used for the rapid testing and development of novel materials, finding its wide applications ranging from nonlinear optics, optical sensors, and atom optics to fiber lasers.

## 3 Conclusions

In conclusion, we presented an improved multi-scan laser direct-writing technique for fabrication of low-loss PLLSW in a glass substrate, based on a single-step inscription method, without additional processes like etching and polishing. The fabricated PLLSW exhibits uniform positive index distribution across the cross-section and a minimum insertion loss of 1 dB. We further demonstrate the fine control of PLLSWs depth over a wide range from 1 μm to 10 μm, which enables the controlling mode leakage out of the waveguide. Based on the PLLSW, we demonstrate on-chip light control by designing an evanescent-field-type SA through coupling the PLLSW with a carbon nanotube thin film, achieving maximum modulation depth and minimum saturation power of 10 % and 22.1 MW/cm^2^, respectively. Furthermore, by leveraging the nonlinear optical response of the waveguide-coupled SA, we develop an optical switch for the generation laser pulses at 1.55 μm through the Q-switching method. In summary, our work demonstrates an effective method to integrate nonlinear optical modulators into a glass chip, achieving light-modulated 3D waveguides, which holds great potential for construction of large-scale programmable photonic circuits in the future.

## Supplementary Material

Supplementary Material Details
