# Supplementary material for: Low-loss skimming waveguides with controllable mode leakage for on-chip saturable absorbers
Source: Nanophotonics. 2023 May 31;12(15):3069–76. doi: 10.1515/nanoph-2023-0049 (PMC11501599; doi:10.1515/nanoph-2023-0049)
Supplement: Supplementary file 1 — Supplementary Material Details [file j_nanoph-2023-0049_suppl_001.docx]

Yi Yang^a^, Lijing Zhong^a,^*, Yudong Cui, Yuying Wang, Daoyuan Chen, Kuen Yao Lau, Xiaofeng Liu, Zhijun Ma*, Giuseppe Barillaro, Zhi Chen*, and Jianrong Qiu*

**Low-loss skimming waveguides with controllable mode leakage for on-chip saturable absorbers**

**Supplementary text**

The coupling loss is calculated based on the overlap integral of the single-mode fiber mode field and the waveguide mode field[1]. The measured near-field distributions as typically shown in Fig. S6 represent the mode intensity distribution *I*_1_(x,y). We confirm that the wave in the waveguide propagates in a single mode because the output mode field does not change with slight movement of the input fiber. Therefore, there is no interface between the multimodes, and the relationship between the electric field distribution *E*_1_(x,y) and mode field distribution *I*_1_(x,y) can be described by the formula as:

$E_{1}(x,y)=\sqrt{I_{1}(x,y)}$. (1)

Based on the mode theory[2-4], the electromagnetic fields E and H propagating in a single-mode waveguide can be can be expressed as the sum of a bound mode (*E_1_*) and an integral over unbound modes (*E_p_*)：

$E(x,y,z)=a_{1}E_{1}(x,y)\times exp(i\beta_{1}z)+\int a_{p}E_{p}(x,y)exp(i\beta(p)z)dp$, (2)

where, *β_1_* and *β* are the z-direction component of bound mode and unbound modes wave vectors respectively, *a_1_* and *a_p_* are their corresponding coupling coefficient. *E_p_* is also known as leaky mode which is divergent in space, and is evanescent at the specific waveguide area. The input electric field can be written as:

$E(x,y,0)=a_{1}E_{1}(x,y)+\int a_{p}E_{p}(x,y)dp$. (3)

Since *E_p_* attenuates rapidly in the waveguide region, the corresponding power loss is regarded as a sort of loss named coupling loss. The coupling loss γ can be written as:

$\gamma=1-\frac{a_{1}^{2}\iint\left| E_{1}(x, y) \right|^{2}\mathrm{dA}}{\iint\left| E(x,y,0) \right|^{2}\mathrm{dA}}$. (4)

Because different modes are orthogonal, the coupling coefficient *a_1_* can be determined by an overlap integral:

$a_{1}=\frac{\iint E_{1}(x,y)\times E(x,y,0)dA}{\iint\left| E_{1}(x,y) \right|^{2}\mathrm{dA}}$. (5)

Thus, the coupling loss can be calculated by:

$\gamma=1-\frac{\left( \iint E_{1}(x,y)\times E(x,y,0)dA \right)^{2}}{\iint\left| E(x,y,0) \right|^{2}dA\times\iint\left| E_{1}(x,y) \right|^{2}\mathrm{dA}}$. (6)

In the experiment, the waveguide is butt-coupled with a single-mode fiber (SMF-28, Corning). The electric field distribution E(x,y,0) of the fiber is measured from the corresponding mode field distribution as in (1). The coupling loss is obtained by numerically calculating (6).

**Supplementary figures**

**
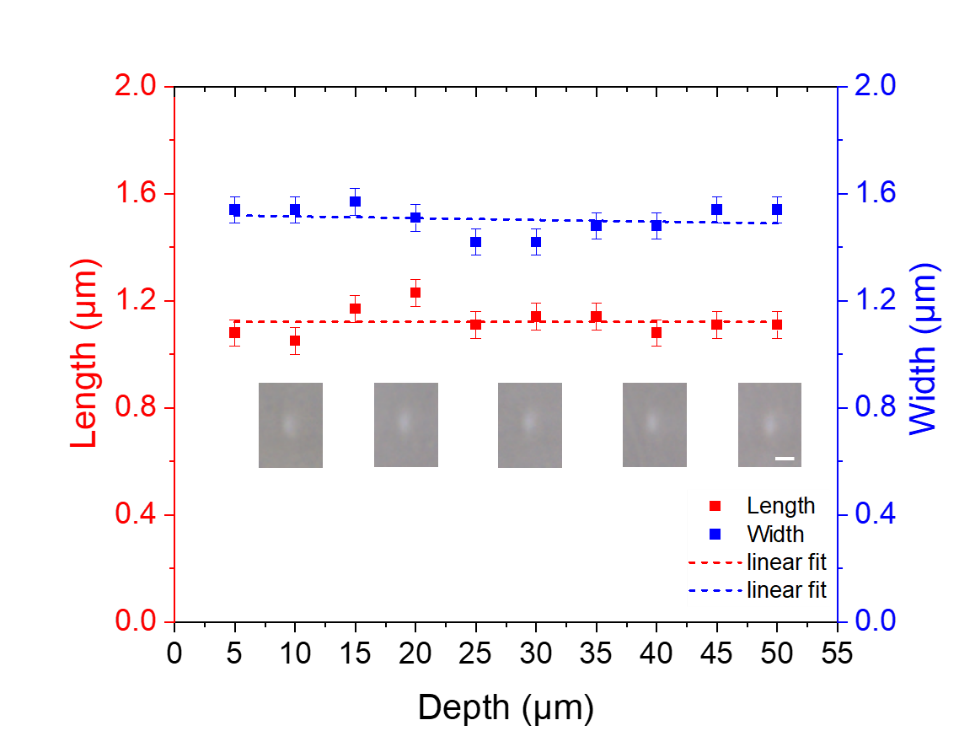
Fig. S1**: Relationship between laser machining depth and the size of the writing area. Illustrations is microscopic cross-section images of tracks at different depths. The scale bars in the bottom right of all images are 1 μm. All the tracks are written by 5 mm/s scanning speed and 16 nJ pulse energy in Eagle XG.

**
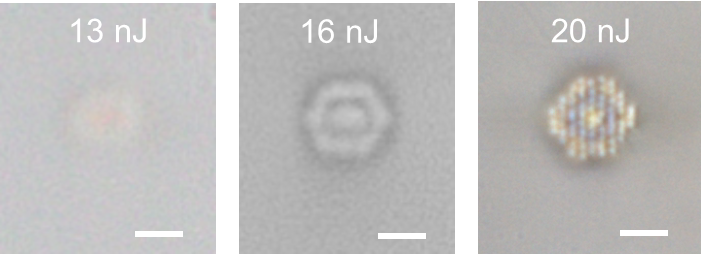
Fig. S2**. Microscopic images of waveguide cross-sections written with laser pulse energies ranging from 13 to 20 nJ. Scale bars are 5 μm.

**
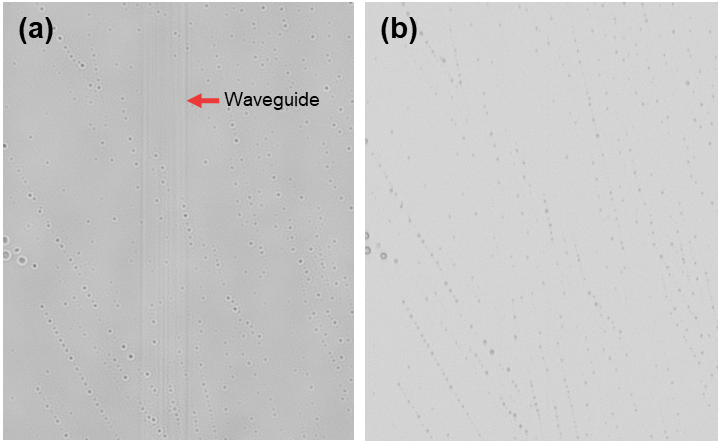
Fig. S3**. Top-view of the glass surface containing a waveguide written to a depth of 1 um with a pulse energy of 16 nJ under transmitted (a) and reflected (b) illumination.

**
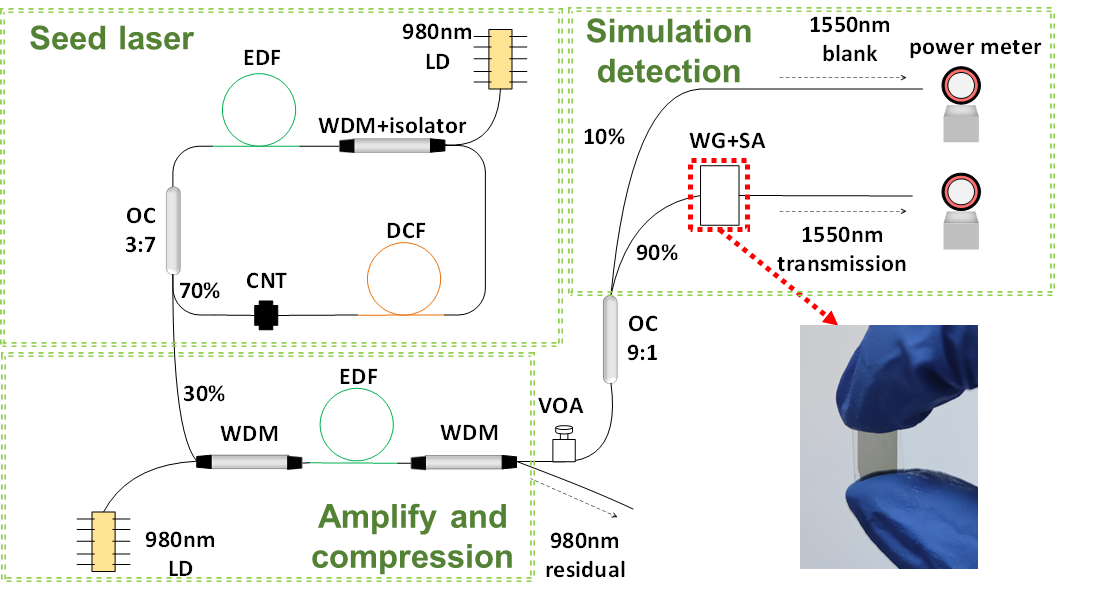
Fig. S4**. Balanced twin-detector measurement system for determining saturable absorption property. Insert, SA consisting of a PLLSW integrated with a SWCNT/PVA film. The waveguides are written along x-axis inside the glass chip.


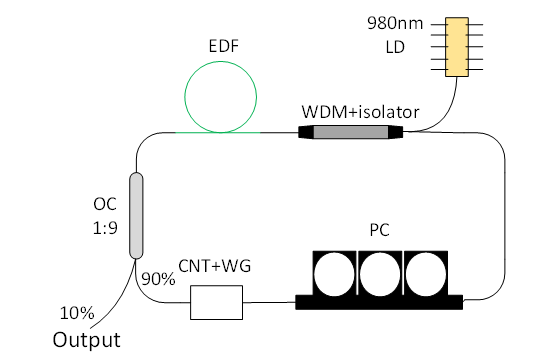


**Fig. S5**: Home-aligned Er^3+^ doped fiber laser used for measurement of the PLLSW waveguide coated with CNT/PMMA film. PC--polarization controller. LD--laser diode. WDM--wave division multiplexer. EDF--Er^3+^ doped fiber. OC--optical coupler. WG+CNT--saturable absorber consist of a PLLSW waveguide coated with CNT/PMMA film.


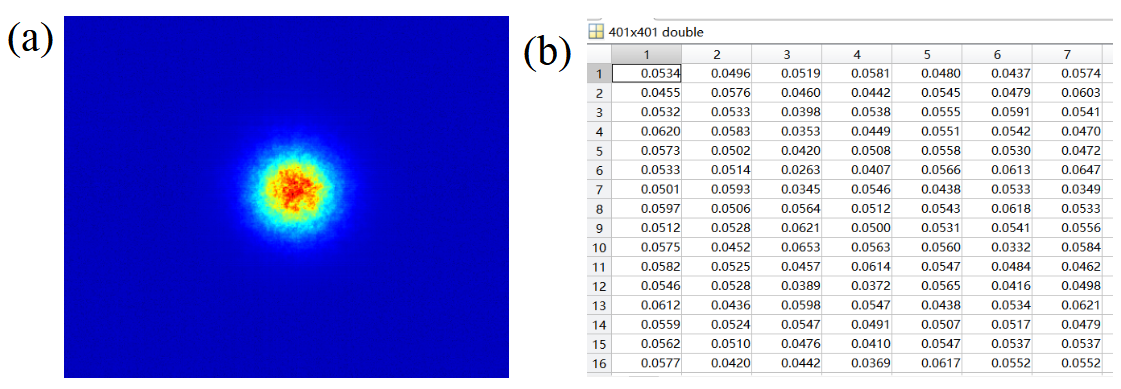
**Fig.S6.** (a) Typical mode field distribution of the waveguide fabricated at 17 μJ and (b) part of corresponding intensity data.

**References**

1. R. E. Wagner and W. J. Tomlinson, "Coupling efficiency of optics in single-mode fiber components," Appl. Opt. **21**, 2671-2688 (1982).

2. A. Bresler, G. Joshi, and N. J. J. o. A. P. Marcuvitz, "Orthogonality properties for modes in passive and active uniform wave guides," **29**, 794-799 (1958).

3. R. Sammut and A. W. J. A. o. Snyder, "Leaky modes on a dielectric waveguide: orthogonality and excitation," **15**, 1040-1044 (1976).

4. D. Marcuse, *Theory of dielectric optical waveguides* (Elsevier, 2013).
